# BUBs Are New Biomarkers of Promoting Tumorigenesis and Affecting Prognosis in Breast Cancer

**DOI:** 10.1155/2022/2760432

**Published:** 2022-04-21

**Authors:** Shunan Wang, Xinyu Liu, Meng Yang, Dongqi Yuan, Kui Ye, Xin Qu, Xinchao Wang

**Affiliations:** ^1^Department of Thyroid and Breast Surgery, Tianjin Fourth Central Hospital, Tianjin 300140, China; ^2^The Fourth Central Clinical School, Tianjin Medical University, Tianjin 300140, China; ^3^Department of Vascular Surgery, Tianjin Fourth Central Hospital, Tianjin 300140, China

## Abstract

**Background:**

A tumor occurs because of abnormal cell multiplication caused by many variables like a significant disturbance in the regulation of cell growth and the instability of chromosome mitosis. Budding uninhibited by benzimidazoles 1 (BUB1), BUB1 mitotic checkpoint serine/threonine kinase B (BUB1B), and budding uninhibited by benzimidazoles 3 (BUB3) are key regulators of mitosis, and their abnormal expression is highly correlated with breast cancer (BrCa), sarcoma, hepatic carcinoma, and other malignant tumors. However, the occurrence of BUBs (BUB1, BUB1B, and BUB3) and the development of BrCa have not been systematically explained.

**Methods:**

Find out the target gene by looking up literature on PubMed and CNKI. Using the R software, TCGA, GEO, Kaplan-Meier Plotter, TIMER, and other databases, we studied the level of transcription, genetic changes, and physiological functions of BUBs in BrCa patients and their relationship with the origin, development, prognosis, immunity, and drug resistance of BrCa patients. *Findings*. We found that the high expression level of BUBs in BrCa tissues proposed a poor prognosis. The multivariate Cox regression analysis suggested that BUB1B and BUB3 might be independent prognostic factors of BrCa. In addition, the Metascape functional enrichment analysis showed that BUBs may be involved in the composition of the spindle, chromosome, and other structures and play a role in mitosis, sister chromatid separation, and other processes. Pathway enrichment suggests that BUBs may affect the cell cycle and lead to abnormal proliferation. Meanwhile, we also found that BUB3 can negatively regulate B lymphocytes, and BUB1 and BUB1B inhibit immune responses by promoting the secretion level of checkpoint molecules of the immune system, leading to immune escape of tumor cells.

**Conclusion:**

We speculate that BUB1, BUB1B, and BUB3 may be therapeutic targets for BrCa patients and also provide new therapeutic strategies for BrCa treatment.

## 1. Introduction

According to the latest global cancer data for 2020, released by the International Agency for Research on Cancer, BrCa has overtaken lung cancer as cancer with the highest number of new cases. What is more worrying is that the incidence of BrCa in China has been increasing year by year in recent years. BrCa has become the primary reason for cancer-associated mortality for females in third-world nations. As with other malignancies, aneuploidy is a common feature of BrCa and affects its behavior. Aneuploidy is related to the inappropriate activity of the spindle assembly checkpoint (SAC). SAC is a monitoring mechanism. When the integrity of the genome is impaired, a sister chromatid separation error during mitosis will lead to aneuploidy, which leads to the occurrence of cancer [1, 2].

When it comes to spindle mitosis, the BUB gene family has important functions to perform. BUB1 is necessary for SAC signaling and correct alignment of chromosomes [3]. As a paralogous gene of BUB1, BUB1B suppresses late anaphase-promoting complex or cyclosome (APC/C) activity via interfering with the binding of the cell division cycle 20 (CDC20) to APC/C and monitors centromere protein E- (CENPE-) dependent kinetochore activity [4]. BUB3 facilitates the establishment of a robust termination on the dipole association, which is required for BUB1 to be localized to the kinetochore. When SAC is activated, the BUB1/3 complex performs its function in inhibiting late APC/C and inhibits APC/C ubiquitin ligase activity by phosphorylating its activator CDC20 [5].

The Genome Project has accumulated a large amount of biological information, and the task of bioinformatics is to mine and utilize this information. Therefore, bioinformatics will play an extremely important role in the “postgenome” era, which will help to understand the information of the human genome. Many cancers, including BrCa, have been deeply explored in terms of genome and transcriptome [6, 7]. Through bioinformatics, the combination of gene expression profiles and clinical data to mine potential biomarkers and provide new targets for cancer treatment has become a hot topic. Therefore, we explored the clinical significance of BUBs in BrCa patients through public databases such as TCGA and GEO, to find new targets for BrCa diagnosis and treatment.

## 2. Materials and Methods

### 2.1. Data Acquisition

The level 3 HTSeq-FPKM format RNAseq data of BrCa was downloaded from TCGA database (https://portal.gdc.cancer.gov/), and a total of 1109 tumor samples and 113 normal samples were obtained. Meanwhile, the clinical information of BrCa patients was downloaded from TCGA database. We found dataset GSE155478 in the GEO database (https://www.ncbi.nlm.nih.gov/geo/) and divided it into two groups according to drug sensitivity: the drug resistance group (3 cases) and the sensitive group (3 cases).

### 2.2. Analyses of Gene Expression

We examine the differential expression of BUBs in BrCa tissues and normal samples in TCGA database by using the R package ggplot2. Then we still use the ggplot2 R package, select the level 3 HTSeq-FPKM format RNAseq data and clinical data in TCGA BrCa project, transform the FPKM format RNAseq data into log2, and examine the relationship between the expression of BUBs and pathological stages. UALCAN is a sophisticated and dynamic web resource (http://ualcan.path.uab.edu/analysis.html) website to detect the differential expression levels of BUBs in different molecular types in BrCa.

### 2.3. Survival Prognostic Analysis

Using the Kaplan-Meier Plotter, researchers can determine whether 54000 genes (mRNA, miRNA, and protein) have an impact on survival in 21 cancer types, notably lung cancer (*n* = 3,452), gastric cancer (*n* = 1,440), ovarian cancer (*n* = 2,190), and breast cancer (*n* = 7,830). The datasets were compiled by data from GEO, EGA, and TCGA. The major goal of the tool is to create and validate survival biomarkers using meta-analytic techniques. First, we select Start KM Plotter for BrCa, then click Use Multiple Genes in the blank column of Gene Symbol to input BUB1, BUB1B, and BUB3 genes, and then select median to divide patients. In the Probe set options, User Selected Probe Set was selected, and OS and RFS were selected successively in Survival to obtain the overall survival (OS) as well as relapse-free survival (RFS) data of BUB transcription in BrCa patients, respectively. To do additional research on the possible prognostic significance of BUBs in BrCa patients, uni- and multivariate Cox regression analyses were performed first, and the forest plot R tool was used to create forest plots to represent the hazard ratio, *p* value, and 95 percent confidence interval for each variable. Because of the multivariate Cox proportional hazard analysis, it was necessary to apply the R software package rms to create a nomogram that could forecast the overall recurrence rate in one, three, and five years. The nomogram provides a graphical representation of these factors, and the prognostic risk of a single patient can be calculated by the points related to each risk factor.

### 2.4. Gene Enrichment Analysis in Relation to BUBs

Using the stat R package, the top 20 genes most associated with BUBs' expression in BrCa were obtained from TCGA database. The duplicate genes were removed, and 45 genes were obtained for further enrichment analysis. The GEPIA online software (http://gepia.cancer-pku.cn/) was used to analyze the correlation between BUB1, BUB1B, and BUB3. BUBs and closely linked genes were investigated by using STRING (http://string-db.org/), which was used to construct a protein interaction network for BUBs and closely associated genes. Subsequently, the Cytoscape software (http://www.cytoscape.org/) was used for visual analysis of the protein-protein interaction (PPI) network. It is more important for the overall stability of the network and if in case the nodes have a higher degree of connection than others. CytoHubba is a Cytoscape plugin that may be used to determine the degree of each protein node in a network of proteins. The top ten genes found in our study were termed “hub genes.” In addition, we used the Metascape (http://metascape.org) website for the Kyoto Encyclopedia of Genes and Genomes (KEGG) and Genetic Ontology (GO) analyses. The latter is composed of three components: biological process (BP), molecular function (MF), and cellular component (CC), and it can be used to predict the functions of genes that are closely associated to BUBs, while the former can describe the gene pathways related to BUBs. Items having a minimum count of 5, a *p* value less than 0.01, and an enrichment factor of enrichment more than 2.0 were collected for further analysis. We use the GeneMANIA (http://genemania.org/) database to construct the BUB interactive network.

### 2.5. Immune-Related Analysis

Using the GSVA package in the R software and GSEA calculation method, we calculated the correlation between BUB1, BUB1B, BUB3, and 24 kinds of common immune cells. Then we use the TIMER (https://cistrome.shinyapps.io/timer/) database, first for the systematic analysis of different cancer types of immune invasion sites, and then choose the Survival module that analyzes the 6 kinds of common immune infiltrating cells in their relationship with prognosis. Then we use the TISIDB (http://cis.hku.hk/TISIDB/index.php) database to further analyze the relations between the BUBs and B lymphocytes; TISIDB is a website of tumor-immune system relationship, and it integrates a variety of data types. To verify the above conclusion again, we picked five typical B lymphocyte gene markers from the Systems website of R&D (https://www.rndsystems.com/cn/resources/cell-markers/immune-cells) and used them in our experiments. The correlation between them and BUBs was analyzed by using the Correlation module of TIMER. Then, we selected seven major immune checkpoint molecules. In TISIDB, the expression level of BUBs was at the *x*-axis, and the abundance of immune checkpoint molecules was at the *y*-axis. It was decided to utilize scatter plots to demonstrate the relationship between the quantity of each immunological checkpoint molecule and the transcription of BUBs.

### 2.6. Drug Resistance Analysis

Using the 45 coexpressed genes, most relevant to BUB expression in BrCa obtained above, and the differentially expressed genes in the GSE155478 dataset related to BrCa resistance found from the GEO database (conforming to critical criteria: adjusted *p* < 0.05 and |logFC| ≥ 1.5 genes are differentially expressed genes) takes the intersection to obtain differential genes coexpressed with BUBs in BrCa resistant cells.

## 3. Results

### 3.1. BUBs Expressed Differentially in BrCa and Normal Breast Tissue

**Figure 1:**
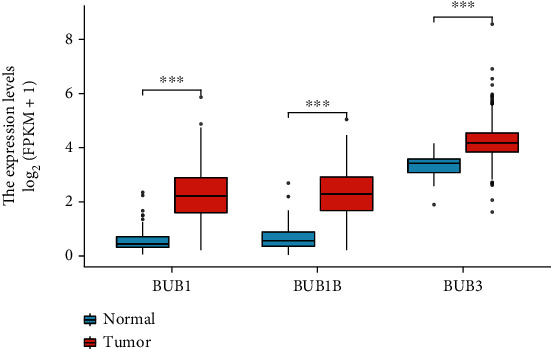
Plot between BrCa and normal breast tissue; there is a difference in the expression of members of the BUB family. ^∗∗∗^*p* < 0.001.

BrCa and normal breast tissues were examined for BUB's expression levels. When compared to normal breast tissue, BrCa tissues had significantly higher levels of BUB1, BUB1B, and BUB3 (Figure 1).

### 3.2. Expression of BUBs in the Clinicopathology of BrCa Patients

**Figure 2:**
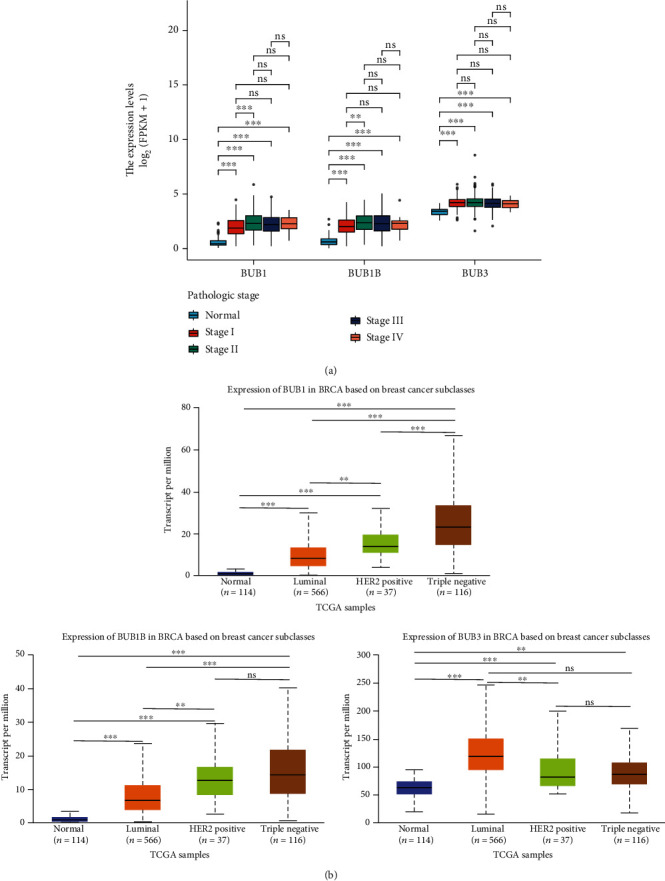
The BrCa clinical stage and molecular type were used to determine the expression of BUBs family members. (a) BUBs are expressed during the clinical stages of BrCa. (b) The expression of BUBs in different molecular types of BrCa. ns, *p* ≥ 0.05; ^∗∗^*p* < 0.01; ^∗∗∗^*p* < 0.001.

After discussing the expression level of BUBs in BrCa and normal tissues, we continued to study the expression of BUBs in BrCa based on cancer in various stages. The expression level of BUBs in BrCa tissues with different pathological stages was higher than that in negative control sample tissues (Figure 2(a)). We further analyzed the expression of BUBs in BrCa patients with different T/N stages. The expression of BUBs in different T/N stages was significantly greater than the level found in negative control tissue samples (Figure S1). In the four common molecular types of BrCa, Her-2 overexpression, luminal (A, B), and three negative breast cancer (TNBC), the expression level of BUBs was higher than that of normal tissues (Figure 2(b)). BUB1 was statistically significant in all genotyping; therefore, the increase in BUB1 expression can infer that the molecular typing is poor. In conclusion, these findings imply that the high level of BUBs may be related to the progress of BrCa.

### 3.3. BUBs' Clinical Prognostic Value in BrCa

**Figure 3:**
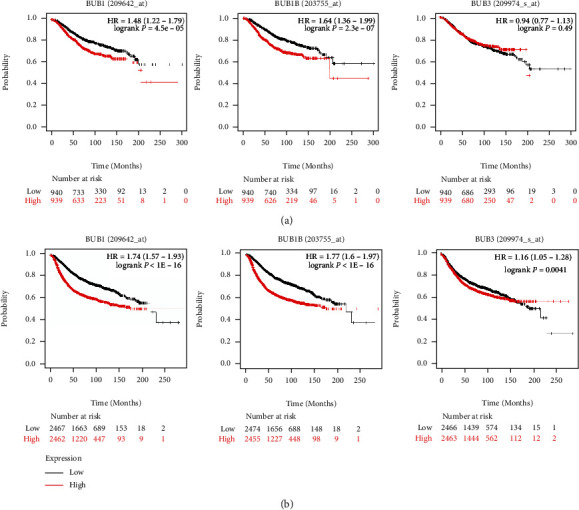
The prognosis of BrCa is affected by BUBs' expression. (a) The effect of BUBs on the OS of patients with BrCa. (b) BUBs and their effect on the RFS of BrCa patients.

To investigate the prognostic significance of BUBs in BrCa, we analyzed the survival data of BrCa patients using the “Kaplan-Meier Plotter database.” We can see the correlation between BUBs differential expression and clinical outcomes of BrCa patients and investigate the prognostic relevance of BUB mRNA expression in patients with BrCa. The high expression of BUB1 and BUB1B resulted in shortened OS and poor prognosis in BrCa patients (Figure 3(a)). The risk ratio of BUB1 was 1.48 (*p* = 4.5*E* − 05), and that of BUB1B was 1.64 (*p* = 2.3*E* − 07), while the expression of BUB3 did not affect the OS rate (*p* = 0.49 > 0.05). The RFS of BrCa patients was significantly and negatively correlated with the expression of BUBs. The risk ratio of BUB1 was 1.74 (*p* < 1*E* − 16), BUB1B was 1.77 (*p* < 1*E* − 16), and BUB3 was 1.16 (*p* = 0.0041) (Figure 3(b)).

### 3.4. Nomograms Predict Survival in BrCa Patients

**Figure 4:**
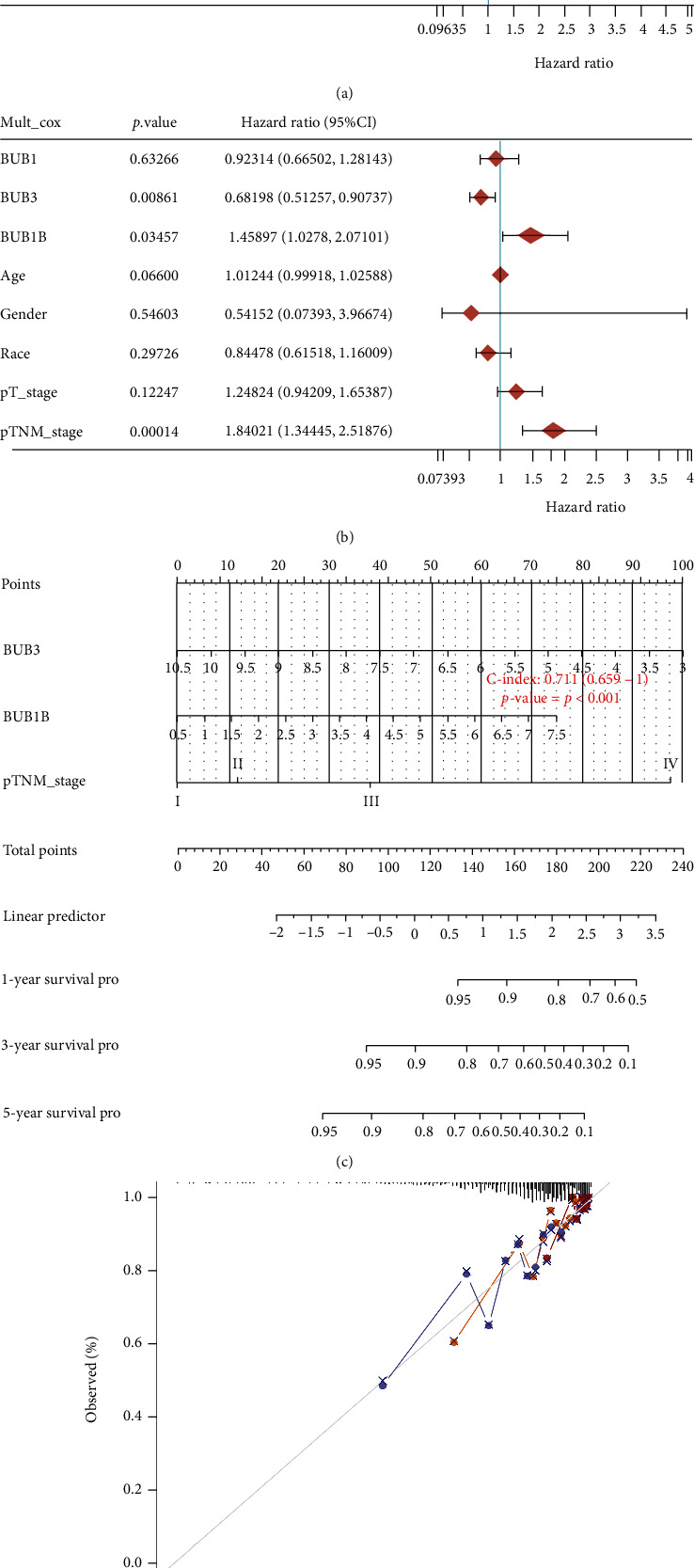
The prognosis of BrCa patients can be predicted using the nomogram model. (a) Regression results from a univariate Cox model are shown in a forest plot. (b) In a forest plot, the results of multivariate Cox regression analysis are displayed. (c) Prediction of BrCa patients' PFS at one, three, and five years using a nomogram. (d) Evaluation of the nomogram model's predictive ability.

In the univariate Cox regression analysis, we found that the high expression of BUB1 (hazard ratio (HR) = 1.16125, 95%confidence interval (CI): 1.00955, 1.33575, *p* = 0.03634) and BUB1B (HR = 1.19911, 95% CI: 1.03011, 1.39585, *p* = 0.01915) suggested that BrCa patients had poor progression-free survival (PFS). The worse T stage (95% CI: 1.3815, 2.05909, HR = 1.6866, *p* < 0.0001) and pathological stage (95% CI: 1.6819, 2.65185, HR = 2.11191, *p* < 0.0001) indicate the worse PFS of BrCa patients (Figure 4(a)). Therefore, BUB1, BUB1B, T stage, and pathological stage are related to PFS. Then, we conducted the multivariate Cox regression analysis and obtained the variables that can be used as nomograms (Figure 4(c)): BUB3 (HR = 0.68198, 95% CI: 0.51257, 0.90737, *p* = 0.00861), BUB1B (HR = 1.45897, 95% CI: 1.0278, 2.07101, *p* = 0.03457), and pathological stages (HR = 1.84021, 95% CI: 1.34445, 2.51876, *p* = 0.00014) (Figure 4(b)). The results of the multivariate Cox regression analysis suggest that BUB1B, BUB3 and pathological stage are variables independent of other clinical factors. However, the difference is that the effect of BUB3 on PFS is opposite to that of BUB1B. A scale is used to indicate the line segment corresponding to each variable in the nomogram, which denotes the variable's value range, and the length of the line segment indicates the factor's prognostic predictive power. The single score is denoted by the point in the illustration. This reflects the unique score assigned to each variable at various levels and then adds the individual scores of each variable to get the total score. According to the total score, the survival rate of the patient in the next 1, 3, and 5 years can be inferred. The more similar the nomogram model to the calibration curve, the more accurate the model's forecast outcome (Figure 4(d)).

### 3.5. Protein Interaction Network and Functional Enrichment Analysis of BUBs and Their Coexpressed Genes

**Figure 5:**
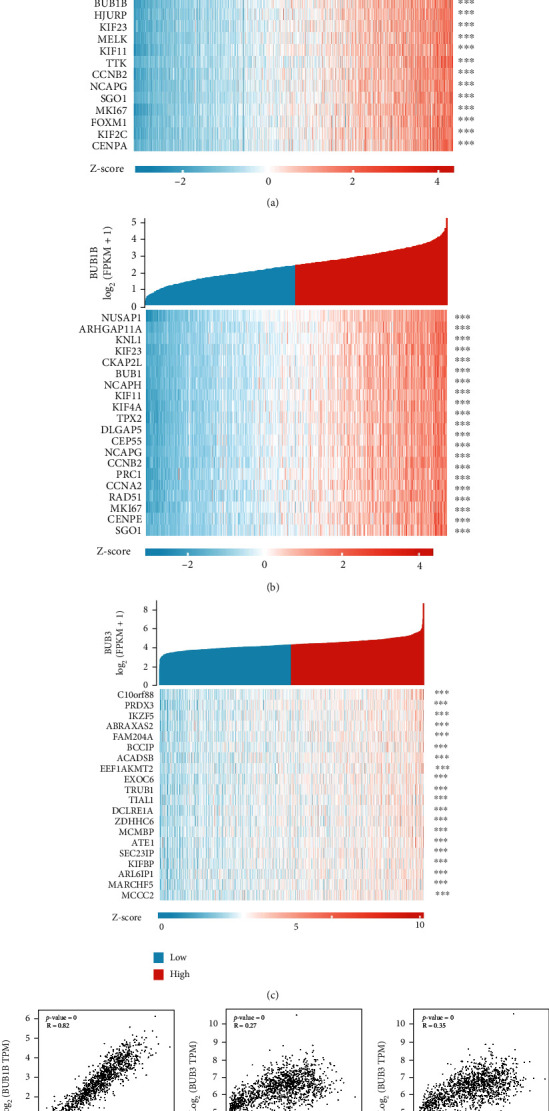
The association between BUBs and coexpressed genes was analyzed. (a) The relationship between BUB1 and the genes that coexpresses. (b) The relationship between BUB1B and the genes that coexpresses. (c) The relationship between BUB3 and the genes that coexpresses. (d) Correlation between BUBs. ^∗∗∗^*p* < 0.001.

**Figure 6:**
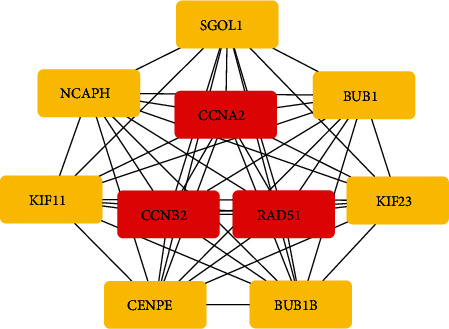
The ten hub genes with the greatest degree of linkage are listed.

TCGA database was used to find out the coexpression genes with BUBs, the correlation coefficients were arranged in descending order, the first 20 genes were selected, and 45 coexpression genes were obtained by removing duplicate genes. We separately studied the correlation between BUBs and their coexpressed genes (Figures 5(a)–5(c)). Additionally, we examined the correlations between the BUBs (Figure 5(d)), which shows that there is a strong correlation among BUB gene families. Then we constructed a PPI protein interaction network between BUBs and 45 coexpressed genes to explore the potential relationship among them (Figure S2) using the CytoHubba plugin in the Cytoscape software; then we identified the top ten hub genes with the highest interactions (Figure 6). It can be seen from the figure that BUB1 and BUB1B are at the core of the complex protein interaction network.

**Figure 7:**
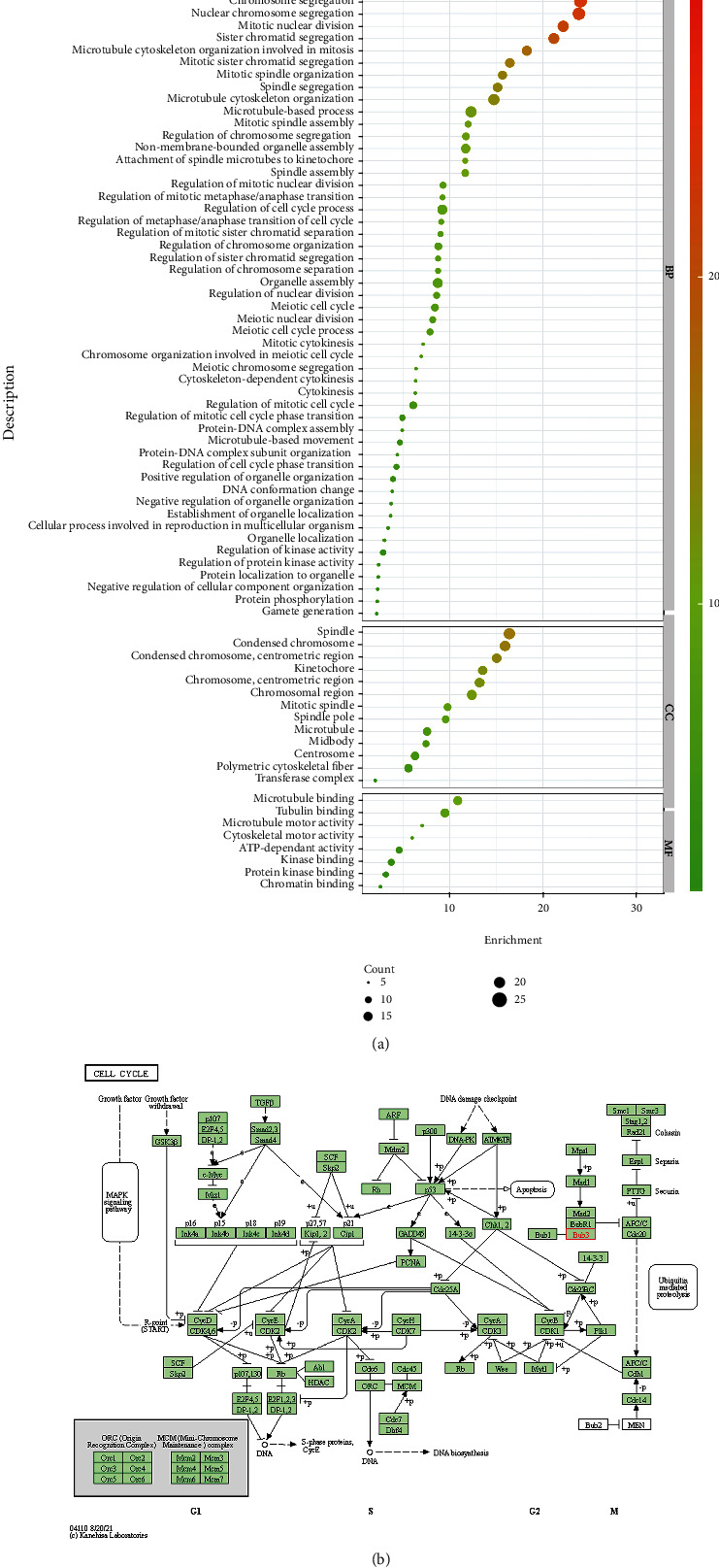
Enrichment analysis using GO and KEGG. (a) GO enrichment analysis. (b) KEGG enrichment analysis.

To further clarify the functional role of the BUB gene family, we used the Metascape online tool to conduct an analysis of the function and enrichment of pathways of BUBs and their closely related genes. The function of BUBs was evaluated from three aspects: BP, MF, and CC (Figure 7(a)). We found that BUBs are mainly involved in the composition of agglutinating the chromosome centromeric region, kinetochore, microtubule, spindle pole, and mitotic spindle and participate in spindle assembly, regulation of mid/late mitotic transition, regulation of chromosome segregation, regulation of sister chromatid segregation in mitosis, cell division, nuclear division, meiotic cell cycle, organelle fission, and other biological processes. It also possesses microtubule binding, ATP-dependent activity, protein kinase binding, and other molecular functions. Pathway enrichment analysis showed that BUBs were involved in the cell cycle pathway (Figure 7(b)). The GeneMANIA database further confirmed the location of the BUB gene family in the chromosome centromere region and in the chromosome region and participates in many processes such as chromosome separation, sister chromatid separation, and mitosis (Figure S3).

### 3.6. Effect of BUB Expression Level on Tumor-Immune Microenvironment (TIME)

**Figure 8:**
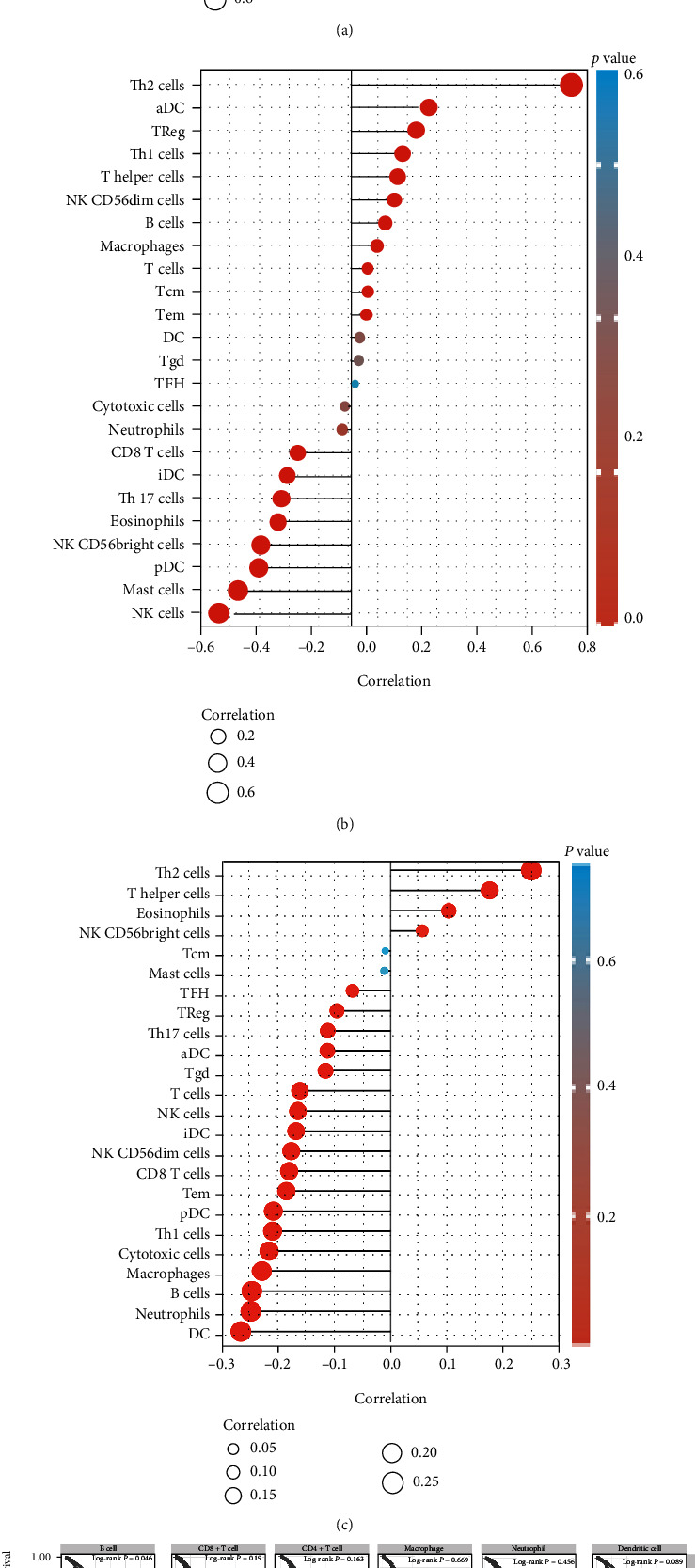
Correlations between BUBs and TILs were analyzed. (a) Correlation between BUB1 and TILs. (b) Correlation between BUB1B and TILs. (c) Correlation between BUB3 and TILs. (d) The effect of six common immune cells on BrCa patient survival. (e) Correlation between BUBs and B lymphocytes. ^∗∗^*p* < 0.01.

Tumor infiltration lymphocyte (TIL) is closely related to the prognosis of BrCa and subsequent immunotherapy [8, 9]. In this study, we investigated the relationship between BUBs expression and immune cell infiltration in the BrCa strain (Figures 8(a)–8(c)). In the case when the correlation coefficient |*ρ*| ≥ 0.2, BUB1 and BUB1B were negatively correlated with mast cells, NK cells, cd56 bright NK cells, eosinophils, plasmacytoid dendritic cells, type 17 T helper cells, and immature dendritic cells, and BUB1 was positively correlated with type 2 T helper cells, activated dendritic cells, regulatory T cells, type 1 T helper cells, and cd56dim natural killer cells; BUB1B was positively correlated with type 2 T helper cells, activated dendritic cells, and regulatory T cells; BUB3 was negatively correlated with dendritic cells, neutrophils, B lymphocytes, macrophages, cytotoxic cells, type 1 T helper cells, and plasmacytoid dendritic cells and positively correlated with type 2 T helper cells. We also used the TIMER to evaluate the impact of six common immune infiltrating cells on the prognosis of BrCa patients (Figure 8(d)). The high expression of B lymphocytes is conducive to enhancing patients' prognosis (*p* = 0.046), and the impact of other immune infiltrating cells on the prognosis is not statistically significant. Therefore, we reverified the link between the degree of expression of BUBs and B lymphocyte infiltration on the TISIDB website (Figure 8(e)). The negative impact of BUB3 on B lymphocytes is greater than the positive impact of BUB1 and BUB1B. BUB3 may affect the prognosis of patients by affecting B lymphocytes. To further explore the relationship between BUBs and B lymphocytes, we found five types of B lymphocytes through the R&D Systems website, selected their surface markers, and analyzed the correlation between BUBs and these surface markers (Table 1). BUB1 and BUB1B had no significant correlation with most markers, but BUB3 had a negative correlation with these markers, indicating that BUB3 negatively regulates B lymphocytes in BrCa. Above, we studied the relationship between BUBs and TILs.

**Figure 9:**
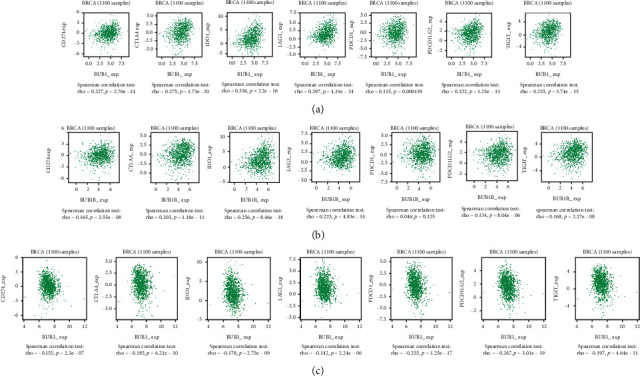
Correlations between the level of BUBs expression and immune checkpoint molecules in BrCa. (a) Correlation between the expression of BUB1 and immune checkpoint molecules in BrCa. (b) Correlation between the expression of BUB1B and immune checkpoint molecules in BrCa. (c) Correlation between the expression of BUB3 and immune checkpoint molecules in BrCa.

However, in the TIME, not only TILs but also immune checkpoint molecules play a role. The immune checkpoint molecule is a regulatory molecule that plays an inhibitory role in the immune system. It is very important to maintain self-tolerance, prevent autoimmune response, and minimize tissue damage by controlling the time and intensity of the immune response. Immune checkpoint molecules expressed on immune cells will obstruct immune cell function, preventing the body from producing an effective antitumor-immune response and promoting tumor-immune escape [10]. To explore whether BUBs have an impact on the expression of immune checkpoint molecules, we selected seven common immune checkpoint molecules for verification in TISIDB (Figures 9(a)–9(c)). BUB1 and BUB1B are positively correlated with these immune checkpoint molecules, while BUB3 is negatively correlated. BUB1 and BUB1B inhibit the immune response by increasing the expression of immune checkpoint molecules, resulting in immune escape of tumor cells, which is conducive to the occurrence and development of BrCa. In conclusion, BUBs affect TIME through different mechanisms, resulting in a poor prognosis of BrCa patients.

### 3.7. Expression of BUBs and Their Coexpressed Genes in BrCa Drug Resistance

**Figure 10:**
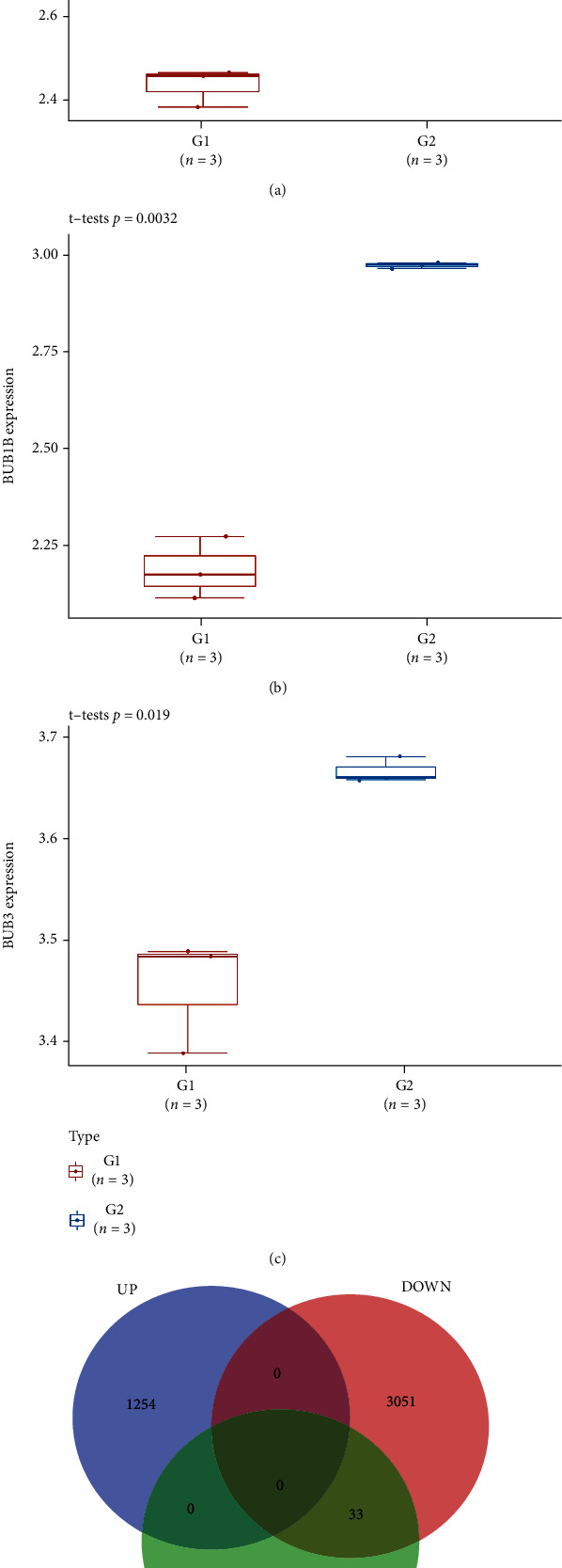
BUBs are expressed differentially in drug-resistant BrCa. (a) The *t*-test was used to determine the difference in BUB1 expression between MCF-7/ADR and MCF-7. (b) The *t*-test was used to determine the difference in BUB1B expression between MCF-7/ADR and MCF-7. (c) The *t*-test was used to determine the difference in BUB3 expression between MCF-7/ADR and MCF-7. (d) The Venn diagram depicts BUBs and their coexpressed genes, as well as GSE155478 differentially expressed genes. G1: BrCa MCF-7/ADR; G2: BrCa MCF-7; UP: GSE155478 upregulated genes; DOWN: GSE155478 downregulated genes.

BrCa is a major public health problem worldwide and is one of the leading causes of death in women. At present, many BrCa treatment drugs have been applied in clinical practice. But long-term use of these drugs can develop resistance, reducing their effectiveness against BrCa and leading to poor survival. In cell development, control of the cell cycle is crucial and plays an important role in the process of tumor drug resistance [9]. Therefore, we searched the GSE155478 dataset in the GEO database to analyze and compare the expression of BUBs and their coexpressed genes in the Adriamycin- (ADR-) sensitive breast cancer cell line MCF-7 and drug-resistant cell line MCF-7/ADR. The *t*-test analyzes the difference in BUB gene expression between BrCa MCF-7/ADR and MCF-7 (Figures 10(a)–10(c)). BUBs and most coexpressed genes showed low expression (Table S1), but no high expression genes, indicating that low expression of BUBs plays a role in the mechanism of drug resistance in BrCa (Figure 10(d)). To avoid cell death, cancer cells have been found to evade drug-induced mitotic arrest (mitotic slip). This mitotic slip is assumed to be the key mechanism for this resistance to drugs [11]. For aneuploid cells to survive, mitotic slippage is required. Changes in the genome composition of aneuploid cells may cause these cells to acquire drug resistance. Chromosomal instability (CIN) is common in solid cancers, mostly due to weakened or overactive mitotic checkpoints. The deteriorated function of the spindle examination point caused by downregulated BUBs expression may lead to mitotic slippage of BrCa cells and then cause acquired drug resistance [12, 13].

## 4. Discussion

SAC is a monitoring system that assists in the appropriate separation of chromosomes during mitosis. Because of less time during mitosis, the chromosomes do not join and separate in the normal manner, resulting in daughter cells with an abnormal number of chromosomes, a state called aneuploidy. Aneuploidy can result in failure to survive, developmental abnormalities, or the initiation and development of diseases such as cancer, depending on its sternness [14–16]. Some studies have shown that aneuploidy induced by SAC damage in the midgut of drosophila will lead to abnormal intestinal development or tumorigenesis [17]. The SAC mechanism is used by cells during mitosis to guarantee that all chromosomes have enough time to attach to spindle microtubules before the cell divides [5, 18]. SAC mainly consists of mitotic arrest defect (MAD) and BUB genes. As a critical regulator of mitosis, BUBs play a critical function in mitotic spindle inspection [19]. BUB1 phosphorylates BUB3 during mitosis, and the BUB1-BUB3 complex acts on APC/C inhibition when the SAC is activated and inhibits APC/C ubiquitin ligase activity by phosphorylating its activator CDC20. Additionally, this compound phosphorylates MAD1L1. In contrast to its requirement for suppressing APC/C^Cdc20^ and chromosome alignment, kinase activity in SAC activity is only a bit important [20]. Even though BUB1's kinase activity is not essential for SAC, it appears that BUB1's primary job is to recruit other SAC components such as MAD1, MAD2, CDC20, and BUB1B to the kinase complex. Additionally, it catalysis the production of the MAD2-CDC20 complex at the centromeres, which eventually forms the mitotic checkpoint complex (MCC), a powerful inhibitor of APC/C^Cdc20^. It is essential for SAC signaling and correct chromosome alignment [3]. BUB1B, a paralogue of BUB1, is likewise necessary for normal mitotic progression. One of its checkpoint functions is to delay the late onset of disease by inhibiting APC/C and ensuring the correct separation of chromosomes. In addition, it is necessary to monitor the activity of kinetochore motors that are dependent on the CENPE kinetochore motor. This protein is required for the localization of the CENPE kinetochore. CENPE interacts with BUB1B, resulting in the activation of its kinase activity, which is important for the activation of SAC. Kinetochore localization of BUB1B is not critical to checkpoint signaling, but it is important to regulate motile microtubule connections. Additionally, BUB1B is important in initiating apoptosis in polyploid cells that retreat abnormally from mitotic stasis, a process that contributes to tumor suppression [2, 21]. Like BUB1, BUB3 locates at the centromere before chromosomal alignment, facilitating kinetochore localization of BUB1, thereby activating checkpoints in response to the unconnected kinetochore. It has dual functions in SAC signal transduction and in establishing proper kinetochore-microtubule attachments [22, 23].

There are reports of BUB gene imbalance in many cancers. For example, BUB1 can enhance the proliferation of hepatocellular carcinoma cells by activating the phosphorylation of SMAD2 [24], and BUB1B promotes liver cancer progression by activating the mechanistic target of rapamycin complex 1 (mTORC1) signaling pathway [25]. Overexpression of BUB1B accelerates the growth of prostatic cancer and predicts a negative prognosis in patients, according to the National Cancer Institute [26]. Overexpression of BUB1 and BUB1B in tumor tissues is related to a poor prognosis in pancreatic ductal adenocarcinoma, and it is also associated with advanced tumor stage and tumor development [27, 28]. Highly expressed BUB3 may weaken the SAC signal by changing the stoichiometric balance of SAC proteins required for MCC formation and checkpoint activity, which may lead to chromosomal missegregation, aneuploidy, and reduced residence time in mitosis [29]. It has also been reported that SAC is essential for the survival of BrCa cells. Inhibition of BUB1B leads to a decrease in the viability and clonogenicity of BrCa cell lines and significantly increases cell apoptosis and cell death. BUB1B gene knockdown can also cause acute chromosomal abnormalities. Knockout of BUB1B on the mouse MDA-MB-468 cell line can reduce tumor growth [2]. Although studies have reported the prognostic effect of BUBs on low-grade BrCa at the protein level [30], BUBs as a gene family interact and are inseparable in the carcinogenesis process. This study analyzed the expression of BUB1, BUB1B, and BUB3 in BrCa from various angles and further explained that the specific BUBs play a role in the incidence and progression of BrCa. We used an online database to compare the differential expression of BUBs in BrCa and normal breast tissues and found that the expression of BUBs in BrCa was higher than that in normal tissues. In addition, Kaplan-Meier Plotter was used to determine a significantly reduced RFS in BrCa patients who overexpressed BUBs, while high expression of BUB1 and BUB1B exhibited a strong correlation with the poorer OS. From the Cox regression analysis on a univariate approach, it is concluded that BUB1 and BUB1B can be used as biomarkers for the prognosis of BrCa. Further analysis of multivariate Cox regression shows that BUB1B and BUB3 may be independent prognostic indicators of BrCa. Immune cells in the body are constantly watching the cells around them, and when they spot alien cells, they activate their immune mechanisms to get rid of the “bad cells” and keep the body safe. It can be seen from the above studies that the high expression of B lymphocytes is beneficial to improving the prognosis of patients, but BUB3 negatively regulates B lymphocytes, making it easy for tumor cells to escape immune surveillance. BUB1 and BUB1B suppress the immune response by increasing the expression of immune checkpoint molecules, causing tumor cells to form immune escape. Studies have found that cells with faulty mitotic checkpoints are more resistant to a variety of anticancer treatments, including microtubule disruptors and DNA disruptors, than cells with normal checkpoints. Mitotic checkpoints are also related to the response to DNA damage. As a result, cancer cells with malfunctioning mitotic checkpoints become resistant to certain anticancer treatments that employ DNA damage as a mechanism of action [31]. We found MCF-7 and MCF-7/ADR in the GEO database and found that BUBs and their coexpression genes were downregulated in drug-resistant cell lines. Antimicrotubular drugs are commonly used as first-line agents for the treatment of a variety of malignancies. In one study, it was suggested that the basis of antiproliferative effects is the result of continuous activation of SAC, prolonged mitotic block, and mitotic cell death caused by microtubule dynamic disturbances. However, cells may also adopt the process of mitotic slippage in addition to mitotic cell death, thereby promoting cell survival and drug resistance [32]. In our study, BUBs are highly expressed in BrCa. Although it cannot be ruled out that the increase in gene expression may lead to instability of the genome, it is more likely to be cell compensation for other molecular component defects in the mitotic spindle damage checkpoint. Increased expression of these genes may be a BrCa marker for chromosomal instability [33, 34]. In the drug resistance analysis, due to the small number of samples, it is necessary to further verify later.

## 5. Conclusion

Through the above analysis, BUBs as biomarkers of BrCa may be potential treatment targets.

## Figures and Tables

**Table 1:** Analysis of correlations between BUBs and a variety of B cell surface markers in BrCa.

B cells	Cell surface markers	BUB1		BUB1B		BUB3	
		Cor	*p* value	Cor	*p* value	Cor	*p* value
Follicular B cell	CD19	0.079	8.54*E* − 03	0.023	4.56*E* − 01	-0.172	9.90*E* − 09
	CD20	0.066	2.81*E* − 02	0.027	3.69*E* − 01	-0.124	3.95*E* − 05
	CD23	-0.058	5.58*E* − 02	-0.09	2.93*E* − 03	-0.221	1.40*E* − 13
Marginal zone B cell	CD1C	-0.155	2.45*E* − 07	-0.176	4.39*E* − 09	-0.142	2.24*E* − 06
	CD19	0.079	8.54*E* − 03	0.023	4.56*E* − 01	-0.172	9.90*E* − 09
	CD21	0.134	8.04*E* − 06	0.076	1.21*E* − 02	-0.185	5.69*E* − 10
Memory B cell	CD40	0.04	1.85*E* − 01	-0.021	4.9*E* − 01	-0.231	8.29*E* − 15
	CD80	0.393	6.08*E* − 42	0.339	5.29*E* − 31	0.078	9.34*E* − 03
	CD21	0.134	8.04*E* − 06	0.076	1.21*E* − 02	-0.185	5.69*E* − 10
Plasma cell	BCMA	0.092	2.24*E* − 03	0.044	1.49*E* − 01	-0.173	7.24*E* − 09
	CD27	0.065	3.04*E* − 02	0.01	7.43*E* − 01	-0.184	8.00*E* − 10
	CD38	0.319	1.63*E* − 27	0.251	2.78*E* − 17	-0.137	5.29*E* − 06
Regulatory B cell	CD1D	-0.097	1.27*E* − 03	-0.113	1.74*E* − 04	-0.178	2.58*E* − 09
	CD21	0.134	8.04*E* − 06	0.076	1.21*E* − 02	-0.185	5.69*E* − 10
	CD38	0.319	1.63*E* − 27	0.251	2.78*E* − 17	-0.137	5.29*E* − 06

Cor: *R* value of Spearman's correlation.

## Data Availability

The datasets of bioinformatics used in this study are available from the corresponding author upon reasonable request.

## Abbreviations

BUB1: Budding uninhibited by benzimidazoles 1
BUB1B: BUB1 mitotic checkpoint serine/threonine kinase B
BUB3: Budding uninhibited by benzimidazoles 3
BUBs: BUB1, BUB1B, and BUB3
BrCa: Breast cancer
SAC: Spindle assembly checkpoint
APC/C: Anaphase-promoting complex or cyclosome
CDC20: Cell division cycle 20
CENPE: Centromere protein E
OS: Overall survival
RFS: Relapse-free survival
CI: Confidence interval
HR: Hazard ratios
PPI network: Protein-protein interaction network
GO: Genetic Ontology
KEGG: Kyoto Encyclopedia of Genes and Genomes
MF: Molecular function
CC: Cellular component
BP: Biological process
TNBC: Three negative breast cancer
PFS: Progression-free survival
TIME: Tumor-immune microenvironment
TIL: Tumor infiltration lymphocyte
ADR: Adriamycin
CIN: Chromosomal instability
MAD: Mitotic arrest defect
MCC: Mitotic checkpoint complex
mTORC1: Mechanistic target of rapamycin complex 1.

## Supplementary Materials

Supplementary 1 Figure S1 The expression of BUBs in different T/N stages of BrCa. ns, *p* ≥ 0.05; ^∗^*p* < 0.05; ^∗∗∗^*p* < 0.001.

Supplementary 2 Figure S2 BUBs and their coexpressed genes protein interaction network.

Supplementary 3 Figure S3 Analysis of BUB gene family in GeneMANIA database.

Supplementary 4 Supplementary Table 1 Differential expression of BUBs and their coexpressed genes in the GSE155478 dataset.
