# Supplementary figures and images for: BUBs Are New Biomarkers of Promoting Tumorigenesis and Affecting Prognosis in Breast Cancer
Source: Dis Markers. 2022 Apr 21;2022:2760432. doi: 10.1155/2022/2760432 (PMC9053761; doi:10.1155/2022/2760432)

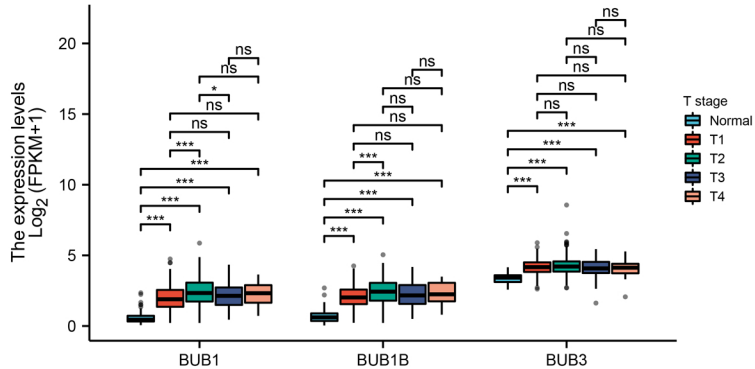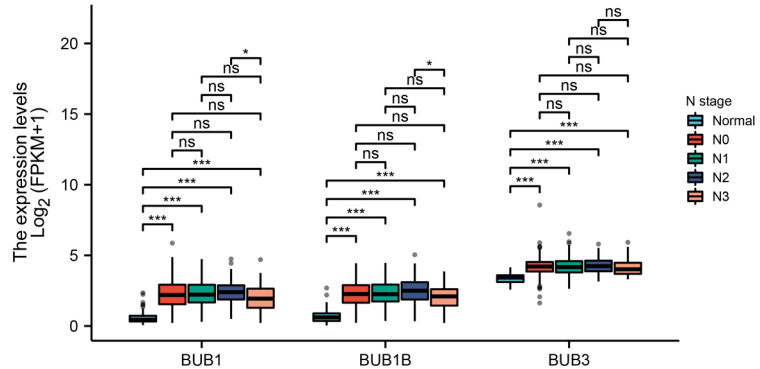

Supplement: Supplementary 1 — Figure S1 The expression of BUBs in different T/N stages of BrCa. ns, p ≥ 0.05; ∗p < 0.05; ∗∗∗p < 0.001. [file 2760432.f1.pdf]

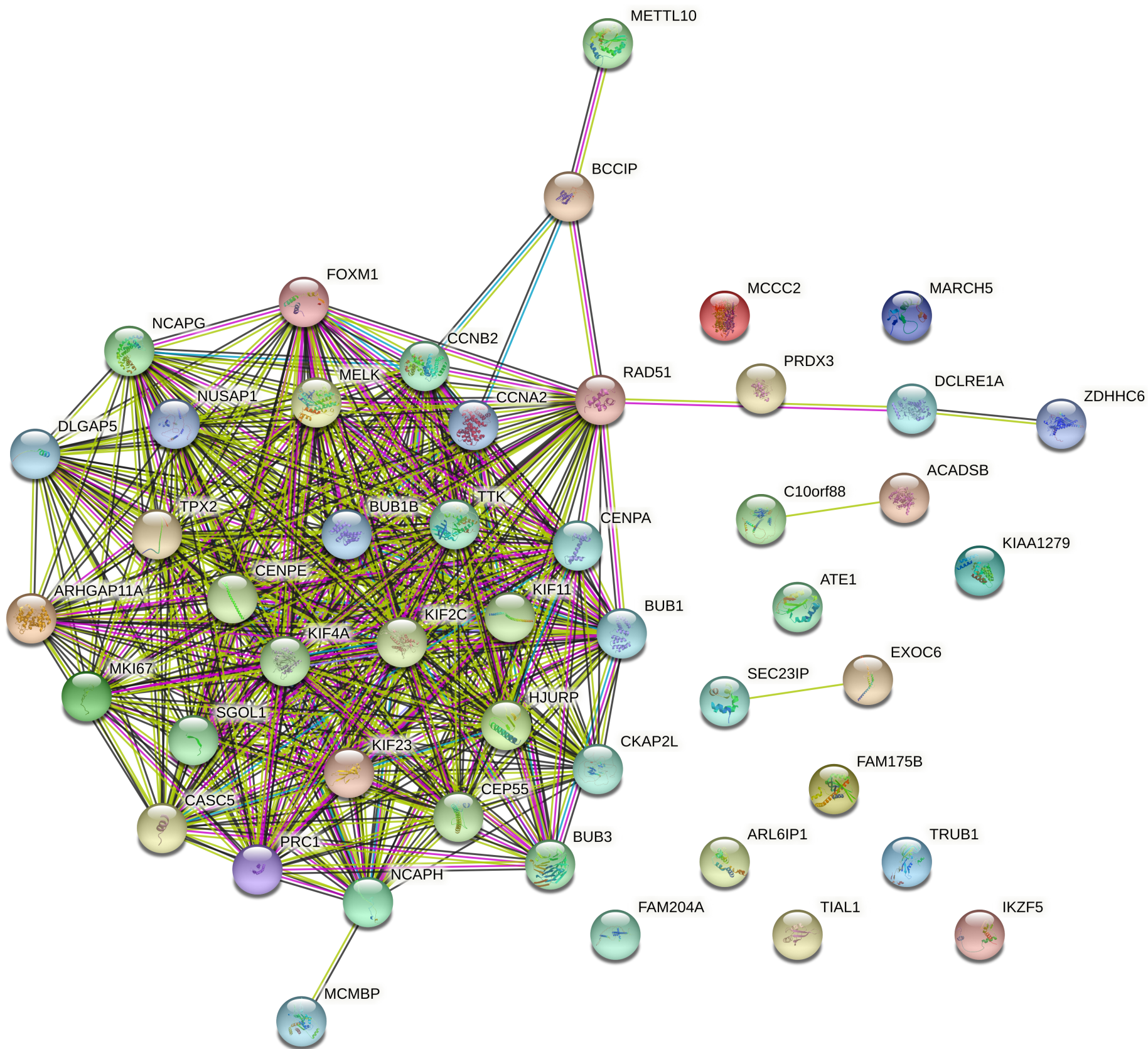

Supplement: Supplementary 2 — Figure S2 BUBs and their coexpressed genes protein interaction network. [file 2760432.f2.pdf]
