# Supplementary material for: BUBs Are New Biomarkers of Promoting Tumorigenesis and Affecting Prognosis in Breast Cancer
Source: Dis Markers. 2022 Apr 21;2022:2760432. doi: 10.1155/2022/2760432 (PMC9053761; doi:10.1155/2022/2760432)
Supplement: Supplementary 3 — Figure S3 Analysis of BUB gene family in GeneMANIA database. [file 2760432.f3.pdf]

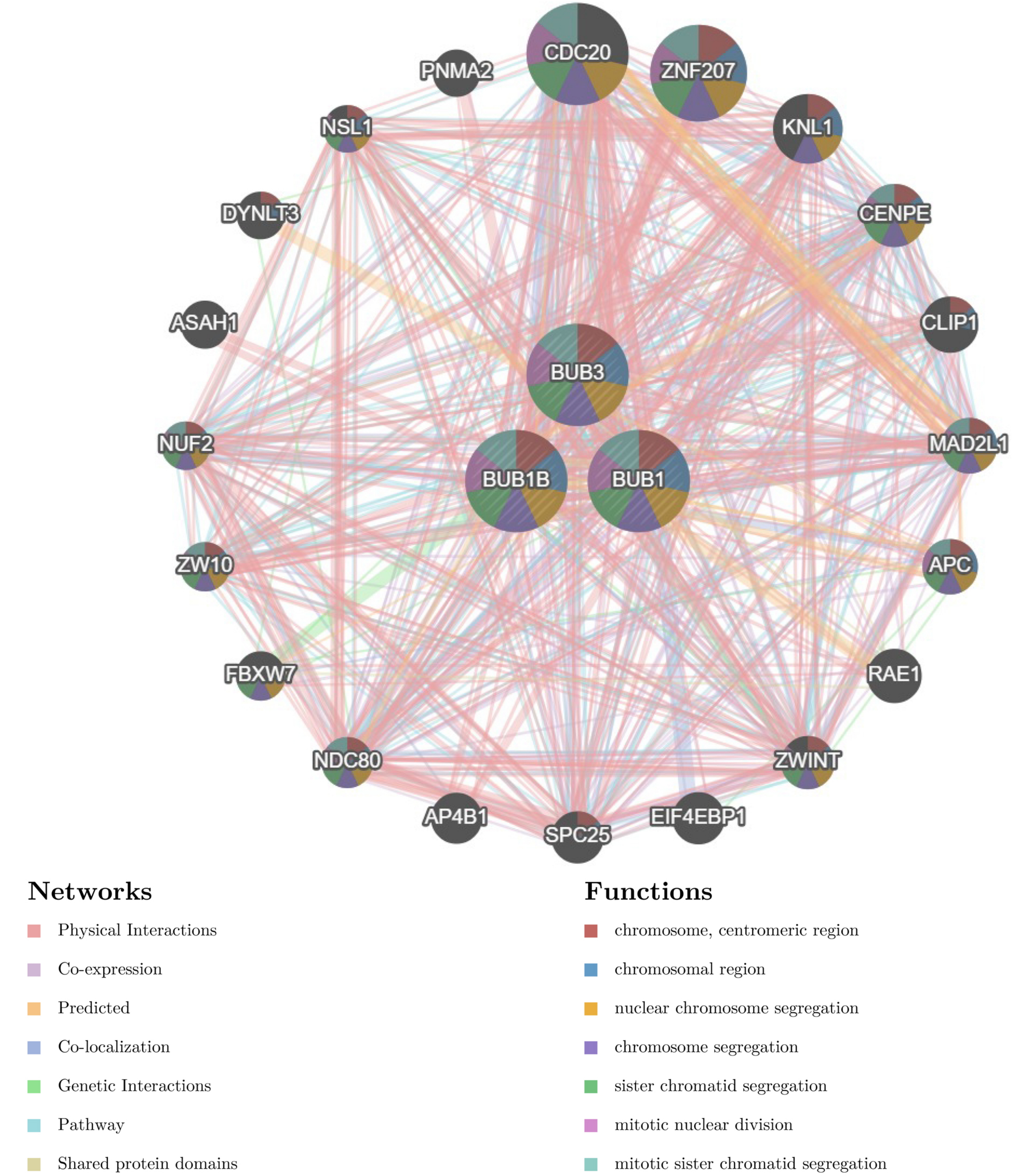
CDC20
ZNF207
PNMA2
NSL1
KNL1
DYNLT3
CENPE
ASAH1
CLIP1
BUB3
NUF2
MAD2L1
BUB1B
BUB1
ZW10
APC
FBXW7
RAE1
NDC80
ZWINT
AP4B1
SPC25
EIF4EBP1
Networks
Physical Interactions
Co-expression
Predicted
Co-localization
Genetic Interactions
Pathway
Shared protein domains
Functions
chromosome, centromeric region
chromosomal region
nuclear chromosome segregation
chromosome segregation
sister chromatid segregation
mitotic nuclear division
mitotic sister chromatid segregation
