# Supplementary material for: BUBs Are New Biomarkers of Promoting Tumorigenesis and Affecting Prognosis in Breast Cancer
Source: Dis Markers. 2022 Apr 21;2022:2760432. doi: 10.1155/2022/2760432 (PMC9053761; doi:10.1155/2022/2760432)
Supplement: Supplementary 4 — Supplementary Table 1 Differential expression of BUBs and their coexpressed genes in the GSE155478 dataset. [file 2760432.f4.pdf]

Table S1 Differential expression of BUBs and their co-expressed genes in the GSE155478 data set

Differential Expression	BUBs and Their Co-expressed Genes
UP	0
DOWN	ARL6IP1; ZDHHC6; FOXM1; KIF4A; MELK; SGO1; CCNA2; KIF23; CCNB2; PRC1; CKAP2L; KIF11; CENPA; BUB1; PRDX3; TPX2; ABRAXAS2; TRUB1; BUB3; CENPE; CEP55; MKI67; MCMBP; ARHGAP11A; IKZF5; KNL1; BUB1B; HJURP; BCCIP; RAD51; TTK; NCAPG; NUSAP1

UP, GSE155478 up-regulated genes; DOWN, GSE155478 down-regulated genes
